# Colorectal Cancer Biomarkers in the Era of Personalized Medicine

**DOI:** 10.3390/jpm9010003

**Published:** 2019-01-14

**Authors:** Jai N. Patel, Mei Ka Fong, Megan Jagosky

**Affiliations:** 1 Department of Cancer Pharmacology, Levine Cancer Institute, Atrium Health, Charlotte, NC 28204, USA; 2 Department of Pharmacy, Levine Cancer Institute, Atrium Health, Charlotte, NC 28204, USA; meika.fong@atriumhealth.org; 3 Department of Solid Tumor Oncology, Levine Cancer Institute, Atrium Health, Charlotte, NC 28204, USA; megan.jagosky@atriumhealth.org

**Keywords:** biomarker, prognostic, predictive, somatic, germline, pharmacogenomics, personalized medicine

## Abstract

The 5-year survival probability for patients with metastatic colorectal cancer has not drastically changed over the last several years, nor has the backbone chemotherapy in first-line disease. Nevertheless, newer targeted therapies and immunotherapies have been approved primarily in the refractory setting, which appears to benefit a small proportion of patients. Until recently, rat sarcoma (*RAS*) mutations remained the only genomic biomarker to assist with therapy selection in metastatic colorectal cancer. Next generation sequencing has unveiled many more potentially powerful predictive genomic markers of therapy response. Importantly, there are also clinical and physiologic predictive or prognostic biomarkers, such as tumor sidedness. Variations in germline pharmacogenomic biomarkers have demonstrated usefulness in determining response or risk of toxicity, which can be critical in defining dose intensity. This review outlines such biomarkers and summarizes their clinical implications on the treatment of colorectal cancer. It is critical that clinicians understand which biomarkers are clinically validated for use in practice and how to act on such test results.

## 1. Introduction

Colorectal cancer is the fourth most common cancer and represents approximately 8% of all new cancer cases in the United States, with over 140,000 estimated new cases in 2018 and 50,000 deaths [1]. Approximately 39% present with localized disease, 35% with regional disease, and 21% diagnosed with metastatic disease. Four percent of cases have an unknown stage at diagnosis [1]. The 5-year survival probability has remained 60–65% from 1996 to 2014, with limited improvements in the last several decades. The 5-year survival for metastatic colorectal cancer (mCRC) is 13.8% [1]. The treatment of colorectal cancer has been built on the backbone of 5-fluorouracil (5-FU) since the 1950s [2]. The standard first-line treatment options for mCRC include 5-FU and leucovorin combined with either oxaliplatin and irinotecan (FOLFOX and FOLFIRI, respectively), plus a monoclonal antibody (bevacizumab versus cetuximab or panitumumab) which is largely dictated by tumor rat sarcoma (*RAS*) status [3]. Targeted therapies in the refractory setting have expanded to include ramucirumab (vascular endothelial growth factor receptor 2 [VEGFR2] inhibitor), aflibercept (vascular endothelial growth factor [VEGF] trapping), regorafenib (tyrosine kinase inhibitor that binds to multiple VEGF-pathway receptors), and immunotherapies such as nivolumab and pembrolizumab that inhibit programmed death 1 (PD1) receptors.

Epidermal growth factor receptor (EGFR) helps regulate cell differentiation and survival via activation of RAS/mitogen-activated protein kinase (MAPK) signaling [4]. Aberrant activation of this pathway can lead to downstream *RAS*, v-Raf murine sarcoma viral oncogene (*RAF*), mitogen-activated protein kinase (*MEK*), and extracellular signal-regulated kinase (*ERK*) phosphorylation and activity, leading to unbridled cell proliferation and oncogenesis. While *RAS* mutations have been the primary gene of interest for monoclonal antibody selection in mCRC, the era of precision medicine and next generation sequencing has unveiled more potentially powerful predictive markers of therapy response. Newer biomarkers, including physiological markers, somatic mutations or immunoprofiling, and germline pharmacogenomics have allowed for better personalization of therapies. This review will focus on the research and clinical application of these biomarkers and implications on the treatment of colorectal cancer. Tables 1–3 summarize key findings and implications of physiologic biomarkers (tumor sidedness), somatic/immune biomarkers, and germline pharmacogenomic biomarkers, respectively.

## 2. Tumor Sidedness as an Independent Predictive and Prognostic Biomarker 

### 2.1. Predictive Biomarker

Right-sided disease arises from the embryonic midgut and encompasses the region from the appendix to hepatic flexure while left-sided disease arises from the embryonic hindgut and includes the region from the splenic flexure to the rectum [5]. Right-sided disease is also more common in older adults and females as opposed to left-sided disease where there is a male predominance. Table 1 summarizes side-specific features in CRC. The importance of tumor sidedness was first noted in 2001 when 5-FU dose optimization studies demonstrated worse outcomes in right-sided disease with a median overall survival (OS) of 10.9 months compared to 15.8 months for left-sided disease [6]. Since then, studies have demonstrated a clear difference in outcomes based on tumor sidedness for mCRC [7,8,9,10]. An analysis of 234 left-sided and 133 right-sided tumors revealed a significant five-year OS difference of 46% and 24%, respectively (*p* < 0.0001) after first-line chemotherapy for mCRC [9]. The OS difference remained significant in multivariate analysis with the addition of biologics, including EGFR- and VEGF-pathway inhibitors.

Large post-hoc analyses have also been performed using cohorts from CALGB/SWOG80405, FIRE-3, CRYSTAL, and TRIBE trials. The CALGB/SWOG80405 trial enrolled 1137 patients to determine whether bevacizumab or cetuximab use in mCRC offered better outcomes when combined with either FOLFIRI or FOLFOX [3]. The selection of biologic therapy did not seem to impact the primary outcome; however, there was a striking difference in median OS between right-sided (19.4 months) versus left-sided tumors (33.0 months). This difference was more pronounced in those who received cetuximab (16.7 vs. 36.0 months; hazard ratio (HR) 1.87, 95% confidence interval (CI) 1.48–2.32; *p* < 0.001). Location origin remained an independent factor after accounting for age, gender, prior chemotherapy, and molecular aberrations (including microsatellite instability (MSI), *BRAF*, *NRAS*, *KRAS*, and *HRAS* mutations) [11].

In the CRYSTAL trial, *KRAS* wild-type patients were randomized to FOLFIRI alone or FOLFIRI with cetuximab and in the FIRE-3 trial, a similar population of patients was randomized to FOLFIRI with cetuximab vs FOLFIRI with bevacizumab [12,13]. In FIRE-3, OS was 38.3 and 18.3 months in left-sided and right-sided tumors, respectively (*p* < 0.0001) when cetuximab was added to FOLFIRI. Bevacizumab added to standard chemotherapy did not have the same amplitude of effect with a median OS of 28.0 vs. 23.0 months in left-sided and right-sided tumors, respectively (*p* = 0.038) [14]. It appeared there may be predictive value in using cetuximab first-line in left-sided tumors with no clear impact imparted on those with right-sided tumors [15]. Further work is ongoing to determine the biological mechanism by which left-sided tumors respond better to anti-EGFR therapy.

The TRIBE study involved 358 patients treated with FOLFOX or FOLFOXIRI plus bevacizumab. Right-sided tumors maintained a shorter OS (23.7 vs. 31.0 months, HR 1.42, *p* = 0.01); however, progression free survival (PFS) was not significantly different (10.2 vs. 11.5 months, *p* = 0.083). After *RAS* and *BRAF* mutation status were accounted for, the impact of tumor sidedness on OS was not significant (HR 1.15; 95% CI 0.90–1.47; *p* = 0.252). This highlights the possibility that FOLFOXIRI with bevacizumab may be used in right-sided tumors regardless of mutation status [16]. 

Prompted by its impact in the metastatic setting, the importance of tumor sidedness in the fully resected stage III setting was also investigated. The PETACC-8 trial involving 1,869 resected stage III tumors identified that right-sided tumors had similar disease-free survival (DFS) to left-sided tumors but had significantly worse survival after relapse (HR 1.54, 95% CI 1.23–1.93; *p* = 0.001). In right-sided tumors with a mutation in *BRAF* or *RAS*, there was an improvement in DFS compared to left-sided tumors, but the opposite was true when right-sided tumors had both *BRAF* and *RAS* wild-type (worse DFS) [17]. It appears tumor mutation status may be more prognostic than tumor-sidedness in the fully-resected stage III setting.

A large surveillance, epidemiology, and end results database demonstrated that 3-year OS was 27% and 16% for left- and right-sided tumors in stage IV disease, and 71% and 62% in stage III disease, respectively. Stage I and II tumors did not demonstrate the same association [18]. A large population-based retrospective study of 6365 patients with early-stage colorectal cancer, 52% of which had right-sided disease, identified that disease laterality was not associated with long-term OS (HR 1.03, 95% CI 0.93–1.14) or cancer-specific survival (HR 1.10, 95% CI 0.97–1.24) after adjusting for other known clinical factors [19]. While tumor sidedness seems to impact response in later stage disease, its impact is not evident in earlier stage disease.

### 2.2. Prognostic Biomarker

The biological effect of sidedness on prognosis remains unclear. It is postulated that the difference in survival could be related to lumen caliber being larger on the right side, with more liquid stool and therefore the less likelihood for obstruction resulting in later presentation [20]. With more research, however, the intricate interplay of molecular and biologic causes has been further expounded. *BRAF* and *EGFR* aberrations with greater MSI occur more commonly on the right side with more *TP53* mutations on the left. *APC* mutations and *HER-2* overexpression occur more commonly in the rectum but laterality has remained impactful even when some of these molecular aberrations (MSI, *BRAF*, *NRAS*, *KRAS*, and *HRAS*) are accounted for [20]. 

Using transcriptomics, molecular subtype classification systems have been developed to better understand differences in tumor behavior. These systems include colon cancer subtypes (CCS) and consensus molecular subtypes (CMS) [21]. Right-sided tumors more commonly demonstrate CMS1 (MSI immune) and CMS3 (metabolic) which carry worse prognoses and are associated with *BRAF* mutations. These subtypes are related to the CCS2 class which is also more common on the right-side. CMS2 (canonical) are more commonly left-sided tumors and are similar to CCS1 class which is commonly found on the left-side with marked chromosomal instability, overactivation of the wingless integrated signaling pathway (WNT), and *TP53*/*KRAS* mutations [22]. In addition, right-sided tumors are more commonly goblet-like and inflammatory with left-sided more commonly enterocyte and transit-amplifying with a similar expression of stem cell-like subtype [23]. 

Lastly, there is location variability in the gut microbiome and when this is disrupted dysbiosis contributes to tumorigenesis [24]. Biofilms make up dense aggregates of bacteria and can be pathogenic [25]. They are related to greater epithelial cell activation and propagation with an increase in the pro-angiogenic interleukin-6 and enhanced downstream activation of signal transducer and activator of transcription 3 with a loss of E-cadherin [26]. Biofilms are present on most right-sided tumors but only identified in roughly 12% of left-sided tumors. In addition, certain bacterial strains have been implicated in more frequent mucosal barrier disruption, inflammation, and tumorigenesis, including enterotoxigenic bactericides fragilis, certain species of *E. coli*, and fusobacteria. The concentration of fusobacterium is location dependent with the right-side demonstrating a greater quantity than the left with a steady increase from rectum to cecum [27]. 

Overall, tumor sidedness impacts both prognoses in advanced disease and is predictive of responsiveness to anti-EGFR therapy. National Comprehensive Cancer Network (NCCN) guidelines now recommend against the use of first-line anti-EGFR therapy for metastatic disease originating from the right-side due to a predictive lack of response [28]. 

## 3. Somatic Mutations and Immunoprofiling

Table 2 summarizes key somatic mutations and their clincial implication in CRC.

### 3.1. RAS 

RAS proteins are proto-oncogenes that are encoded by the *RAS* family genes comprised of *HRAS*, *KRAS* (including isoforms *KRAS4A* and *KRAS4B*) and *NRAS* genes. These genes encode GTPase, a protein that splices GTP to GDP which then turns *RAS* off and stops cell proliferation. The most widely recognized mutations occur at exon 2 codons 12 and 13, which leads to a constitutively active state and ongoing cell expansion. *KRAS* mutations are the predominant mutations found in the *RAS* family genes in mCRC (85%) as compared to *NRAS* (11%) and *HRAS* (4%) [29]. 

Mutated *KRAS* in mCRC is a predictive biomarker for lack of response to EGFR inhibitor antibodies cetuximab and panitumumab [29]. The CRYSTAL trial demonstrated cetuximab added to FOLFIRI in the mCRC first-line setting was less beneficial and perhaps even detrimental among the *KRAS* mutated population compared to FOLFIRI alone [12]. The *KRAS* wild-type tumor group had a 16% increase in overall response rate (ORR) to 60% when cetuximab was added to FOLFIRI but there was a 4% decrease in response to 36.2% among *KRAS* mutated tumors. OS improved from 21.0 to 24.9 months in the *KRAS* wild-type group but did not differ in the *KRAS* mutated population. While the ORR was statistically significant (OR 1.91; 95% CI 1.24–2.93; *p* = 0.03), survival was not statistically significant (HR 0.84; 95% CI, 0.64–1.11). The OPUS trial using cetuximab with FOLFOX in the same setting showed comparable results. *KRAS* mutated tumors were less likely to respond to cetuximab and there was a higher risk of disease progression with a trend for worse survival [30]. The same association was noted with panitumumab plus standard chemotherapy [31]. 

*KRAS* mutations are also independent predictors for worse OS in patients with mCRC undergoing metastectomy of hepatic disease. A study by Margonis et al. showed that patients with *KRAS* mutated mCRC had a worse OS after hepatic surgery than patients with *KRAS* wild-type (HR 1.99; 95% CI 1.21–3.26; *p* = 0.007) [32]. Comparable results were seen when recurrence-free survival (RFS) was assessed in univariate analysis (HR, 1.53; 95% CI 0.99–2.36, *p* = 0.057) and multivariate analysis (HR 1.68; 95% CI 1.04–2.70; *p* = 0.034). Median RFS was 11.8 months and 20.8 months in the *KRAS* mutated and wild-type groups, respectively [32].

Presence of *RAS* mutations in resected stage II and III disease have also been associated with higher rates of recurrent metastatic disease. A study identified higher frequencies of metastatic lung (62%) and brain (56.5%) recurrence when compared to patients with *RAS* wild-type tumors (*p* < 0.001 and *p* = 0.004, respectively) [33]. 

More studies are needed to determine the predictive value of *RAS* mutation status in non-metastatic primary disease and the potential sites of recurrence. Nonetheless, the NCCN [28], American Society of Clinical Oncology (ASCO) [34] and the European Society of Medical Oncology (ESMO) [35] guidelines all recommend the use of extended *RAS* testing as a predictive biomarker prior to administration of EGFR inhibitors in mCRC. Extended *RAS* analysis should include testing of both *KRAS* and *NRAS* exons 2 (codons 12 and 13), 3 (codons 59 and 61), and 4 (codons 117 and 146).

### 3.2. BRAF 

*BRAF* is a proto-oncogene that codes for proteins aiding in the communication of chemical signals from outside the cell to the nucleus through the RAS/MAPK pathway. The most common *BRAF* mutation in mCRC is a glutamic acid substitution for valine at codon 600 (V600E) resulting in activation of *BRAF* and constitutive activation of the MAPK pathway. The *BRAF^V600E^* mutation occurs in 8–12% of mCRC and is mutually exclusive of *RAS* mutations [36,37]. Presence of a *BRAF* mutation results in a poor prognosis with median OS less than 6 months after the failure of first-line mCRC therapy [38,39]. Since *BRAF* is downstream of EGFR, it is suggested that the administration of EGFR inhibitors would exhibit a lack of efficacy for *BRAF*-mutated disease [40]. Conversely, a meta-analysis did not show the same finding. In the meta-analysis, the test of interaction amongst seven randomized clinical trials was not statistically significant for differences in OS between *RAS* wild-type/*BRAF* mutant and *RAS* wild-type/*BRAF* wild-type tumors (*p* = 0.43) [41]. The data, therefore, supporting the predictive capacity of *BRAF* are mixed.

Despite marked success in *BRAF*-mutated melanoma and pre-clinical trials in *BRAF* mutant colorectal cell lines, *BRAF* inhibition has not shown clinical benefit in *BRAF*-mutated mCRC. Single-agent inhibition has demonstrated a PFS of 2.1 months and ORR of approximately 5% with dual RAF/MEK blockade [42]. Preclinical studies suggest reactivation of a compensatory EGFR feedback resulting in continued cell proliferation in the presence of *BRAF^V600E^* inhibition [43,44]. The Southwest Oncology Group S1406 study investigated irinotecan and cetuximab with or without BRAF inhibitor vemurafenib in *BRAF* mutation positive mCRC. One hundred and six patients with mutated *BRAF^V600E^* and RAS wild-type were randomized to irinotecan and cetuximab (doublet therapy) or irinotecan/cetuximab/vemurafenib (triplet therapy) after progression on 1 or 2 prior lines of therapy with no anti-EGFR agents. PFS increased to 4.4 months with triplet therapy compared to 2 months with the doublet (HR 0.42, 95% CI 0.26–0.66; *p* = 0.0002). In addition, triplet therapy resulted in a disease control rate of 67% compared to 22% in the standard-of-care arm [45]. 

The use of *BRAF* as a predictive biomarker for mCRC is limited at this time and the use of single agent *BRAF* inhibitors does not demonstrate the same effects as seen in melanoma. *BRAF* mutational status may be useful in determining EGFR inhibitor treatment with or without BRAF inhibitors. The presence of *BRAF^V600E^* mutation in *RAS* wild-type tumors may theoretically demonstrate a lack of efficacy, but more studies are needed to confirm this theory.

### 3.3. MEK 

MEK is an enzyme downstream of *RAS* and *RAF* which when activated, triggers ERK, the last substrate in the MAPK/ERK pathway which leads to gene transcription and cell growth. Despite upstream *RAS* wild type, the use of an EGFR inhibitor in the presence of *MEK* mutated mCRC is not likely to result in a clinical response. A study by Corcoran et al. used a combination of BRAF inhibitor dabrafenib, MEK inhibitor trametinib, and EGFR inhibitor panitumumab, which reported an ORR of 21% [44]. In the safety lead-in BEACON CRC trial triple inhibition using a BRAF inhibitor encorafenib, MEK inhibitor binimetinib, and EGFR inhibitor cetuximab was given to *BRAF^V600E^* mutation positive patients who had progressed on 1 or 2 prior lines of therapies. The ORR was 48% and median PFS was 8.0 months, which was substantially improved compared to the historical PFS of 2.0 months [46]. The phase III BEACON CRC study is currently ongoing and investigating encorafenib/binimetinib/cetuximab versus encorafenib/cetuximab versus control arm FOLFIRI (or irinotecan)/cetuximab with a primary endpoint of OS (ClinicalTrials.Gov NCT02928224). 

A similar pathway parallel to the EGFR-RAS-MEK pathway that triggers cell growth and tumorigenesis is the WNT signaling pathway. In preclinical trials, MEK inhibitor-resistant *KRAS*-mutated CRC cell lines showed aberrant overexpression of the WNT signaling pathway [47]. Cyclosporin A can inhibit the WNT signaling pathway by inhibiting WNT5a causing apoptosis. In preclinical models, the combination of selumetinib and cyclosporin A showed antitumor activity in selumetinib-resistant *KRAS*-mutant CRC cells [48]. A phase Ib study using a combination of MEK inhibitor selumetinib and calcineurin inhibitor cyclosporin A aimed to identify dosing parameters and to validate the previous findings of WNT pathway involvement. However, the sample size was too small to determine whether there was adequate cyclosporin A suppression of the WNT pathway at the molecular level [49]. 

*MEK* is a potential predictive marker in the presence of *BRAF* mutation. The data to suggest *MEK* involvement in the WNT signaling pathway may provide other treatment options in the future. The prognostic value of *MEK* is unknown at this time. More studies are needed to support the use of *MEK* status as a predictive biomarker and no recommendations are currently available for this purpose.

### 3.4. HER-2 

The *ERBB2* gene encodes for HER-2 (human epidermal growth factor receptor 2) and the presence of sequence mutations may result in altered *HER-2* amplification. The incidence of *HER-2* gene amplification in mCRC is approximately 3%, and slightly higher (5%) in *RAS* wild-type tumors [50,51]. 

The HERACLES-A phase II trial investigated the use of combination lapatinib and trastuzumab in 33 patients with *KRAS* wild-type, *HER-2* amplified mCRC that had progressed on conventional treatment options including cetuximab or panitumumab. The clinical benefit rate was 70% with an ORR of 30.3% at the median 2-year follow-up. The median time to progression was 5.5 months [52]. HERACLES-B is an active trial evaluating pertuzumab with ado-trastuzumab emtansine and HERACLES RESCUE is an ongoing study of ado-trastuzumab emtansine monotherapy in patients who have progressed on lapatinib and trastuzumab in HERACLES A (Clinicaltrials.gov NCT03418558). The ongoing umbrella study, MyPathway, is investigating various solid tumors with actionable mutations in *HER-2*, *EGFR*, *BRAF* or the Hedgehog pathway in which targeted therapy exists. In the gastrointestinal cohort, 16 mCRC patients with *HER-2* amplification receiving trastuzumab and pertuzumab reported an ORR of 37.5%. The median time to progression was 5.6 months [53].

The use of *HER-2* as a prognostic factor is currently unknown, however, preliminary findings demonstrate a potential predictive biomarker for refractory *HER-2* positive mCRC. The modest sample sizes of both studies and non-randomized study design makes it difficult to draw any firm conclusions regarding the use of dual *HER-2* inhibition in mCRC at this time. No guidelines currently recommend *HER-2* testing in mCRC and larger prospective studies of single and combination HER-2 targeted therapies are required prior to adoption. 

### 3.5. PIK3CA 

*PIK3CA* (Phosphatidylinositol-4,5-bisphosphate 3-kinase catalytic subunit alpha) is a gene encoding the p110 alpha catalytic subunit of PI3K (phosphatidylinositol 3-kinase), an enzyme involved in the PI3K/AKT (protein kinase B)/mTOR (mechanistic target of rapamycin) pathway [54]. The *PIK3CA* mutations in exon 9 and/or 20 is found in approximately 10–15% of CRC. In the presence of the mutated *PIK3CA*, the constitutive activation of PI3K results in downstream AKT activation and upregulation of Prostaglandin-Endoperoxide Synthase 2 (PTGS2) and prostaglandin E2 [54]. This mutation may be a predictive biomarker for treatment response to aspirin in patients with CRC. In the multivariate analysis of the VICTOR study (a randomized trial comparing rofecoxib with placebo after primary CRC resection), stage II and III CRC patients with a *PIK3CA* mutation had a lower RFS when taking aspirin 100 mg daily (HR 0.11; 95% CI 0.001 to 0.832, *p* = 0.036) in the post-treatment setting [54]. Another study demonstrated superior cancer-specific survival (HR 0.18; 95% CI 0.06 to 0.61; *p* < 0.001) when aspirin was used after CRC diagnosis in tumors with PIK3CA mutation. In addition, the effect of aspirin was strongest in those with the *PIK3CA* mutation and *PTGS2* amplification, although this was part of an exploratory analysis with a limited sample size [55]. In the metastatic setting, the use of triple inhibition of BRAF, EGFR, and PI3KCA in mCRC is currently being evaluated. In a phase 1b escalation study, triple inhibition using encorafenib, cetuximab, and a PIK3a inhibitor alpelisib showed tolerability and potential for clinical activity (Clinicaltrials.gov NCT01719380) [56]. 

### 3.6. MMR/MSI 

Mismatch repair (MMR) is an enzymatic process that recognizes and repairs damaged DNA. Regions of repeating DNA that are prone to error are known as microsatellites. Mutations in MMR genes including *MLH1*, *PMS1*, *PMS2, MSH6*, and *MSH2* lead to deficient MMR (dMMR). *PMS2* and *MSH6* make up approximately 30% and *MSH2* make sup approximately 70% of the MMR mutations [57]. Tumors that have dMMR are associated with high microsatellite instability (MSI). In stage II and III disease, 15% of CRC have dMMR whereas, in stage IV disease, only 4–5% of tumors have dMMR [58]. The prognostic implications of dMMR/MSI among CRC patients are variable by stage. In stage II and III diseases there is a survival benefit in dMMR/MSI high tumors. In the metastatic setting, the presence of dMMR/MSI has negative prognostic implications, although it could be the concomitant presence of BRAF mutations that promote this finding [38]. In a study of 3063 patients, BRAF mutations were observed in 35% of patients with dMMR tumors compared to 6.8% of patients with proficient MMR (pMMR) (*p* < 0.001) [38]. 

In a prospective study by Sargent et al [59], 457 stage II/III colon cancer tumor specimens were analyzed and approximately 15% were found to have dMMR. Patients with dMMR more commonly had poorly differentiated histology (*p* = 0.002) and were more frequently diagnosed with stage II disease (*p* = 0.006). Surgical intervention alone improved DFS in the univariate analysis with a trend for a better OS, but this was not maintained in the multivariate analysis. There was no DFS or OS improvement in patients treated with 5-FU. dMMR tumors had improved DFS in stage II patients who received surgery alone versus adjuvant 5-FU based chemotherapy (HR 2.30; 95% CI 0.84 to 6.24; *p* = 0.09). In contrast, stage III patients with pMMR tumors derived a benefit from adjuvant 5-FU based chemotherapy (HR 0.64; *p* = 0.001) [59]. This supports the notion that chemotherapy may be avoided in stage II disease where dMMR has an improved prognosis and no benefit from 5-FU based adjuvant therapy. 

The predictive value of MMR status is limited due to the retrospective nature of the data. While the presence of dMMR/MSI may be considered, other factors for high risk disease such as adequate lymph node sampling [60], invasion of visceral peritoneum or penetration/adherence to other organs or structures [61], presence of obstruction or perforation [62], and lymphovascular or perineural invasion [63,64,65] should be considered when deciding on appropriate adjuvant therapy in stage II colon cancer. 

dMMR may also have a predictive role in the use of immunotherapy in mCRC. Somatic mutations associated with dMMR have shown lymphocyte infiltrate, which suggests an immune response in dMMR cancer cells [66]. The expression of PD-1 receptors may be an actionable target in mCRC for anti-PD1 agents such as pembrolizumab and nivolumab. A phase II study evaluated the use of pembrolizumab in mCRC displaying dMMR from hereditary nonpolyposis or somatic/sporadic gene mutations [58]. In tumors displaying dMMR, ORR was 40% with a PFS of 78% at 20 weeks, while OS was not yet reached. Patients with pMMR did not show a response with immunotherapy [67]. In a separate phase 2 study, dMMR/MSI high patients treated with nivolumab and low dose ipilimumab had an ORR of 54.6%. Disease control greater than 12 weeks was reached in 80% of patients [68]. These studies suggest the use of MMR status as a possible predictive marker for immunotherapy agents. NCCN currently recommends MSI testing for all patients with mCRC. Both nivolumab and pembrolizumab are currently FDA approved for use in dMMR/MSI high expressing mCRC.

### 3.7. Tumor Infiltrating Lymphocytes

Tumor infiltrating lymphocytes (TILs) which are found in the stroma, peritumoral, and intratumoral areas, provide a defense mechanism to tumor invasion and their presence has been associated with less blood, lymph vessel, and perineural invasion as well as decreased lymph node involvement [69]. As a result, the adaptive immune system can have a major impact on tumor progression and the response to various treatment modalities including chemotherapy, radiation, and immunotherapy.

The prognostic value of TILs has been demonstrated among different tumor stages in CRC. In early stage I or II CRC, studies have shown improved outcomes with less disease recurrence and greater survival as the quantity of intratumoral cytotoxic and memory T cells increased [70]. Among patients with high-risk stage II or III CRC who have undergone curative resection, a higher quantity of lymphocytes also predicted a better OS and DFS [71,72]. In the mCRC setting, a retrospective analysis of 57 patients’ tumors revealed that high TIL density was associated with a greater response to palliative chemotherapy (ORR 79.3% in high TIL vs. 48.1% in low TIL), PFS, and OS. The palliative chemotherapy regimens varied, but the improved response indicates a predictive component of measuring TILs. The combination of TILs and MSI status may be beneficial in prognosticating disease for individual patients. Of the 150 patient samples, those with MSI−/TIL− disease (less than 5 lymphocytes per 10 high-power fields) had a 5-year DFS of 56.7% in comparison to 88.9% for MSI+/TIL+ tumors [73].

A major limitation of TILs is the variability in methods by which density is evaluated. The International TILs Working Group has helped determine how best to evaluate and use this biomarker, proposing a more stringent, objective method of using H-E-stained samples. They recommend stromal TILs be quantified preferentially above intratumor and peritumoral TILs since the counts are more reliable, reproducible, and can be a consistent variable compared amongst studies [74]. Given the lack of guidelines, clinical application of TILs to guide therapy in mCRC remains limited; however, with the growing use of immunotherapies, improved diagnostic tests, and initiatives set forth by the Working Group, the use of TILs in mCRC management may increase in the coming years. 

### 3.8. POLE Mutations

A newer, albeit rare, biomarker that has been investigated is the presence of exonuclease domain mutations in the polymerase epsilon, catalytic subunit (*POLE*) gene. It was discovered when The Cancer Genome Atlas discovered a population of tumors that were hypermutated but microsatellite stable (MSS). The *POLE* mutations lead to a deficiency in proofreading activity, therefore, errors in DNA replication occur with a greater burden of mutations [75]. The use of *POLE* as a biomarker has been investigated to a greater degree in other tumor types including endometrial, where their presence is associated with more TILs and a greater capacity for immunotherapeutic response [76]. The incidence of these mutations is variable (0.65%–12.3%) among CRC depending on publication due to sensitivity differences in genome sequencing assays [77,78].

Domingo et al. found these mutations to be most prevalent in males, younger patients, and more commonly right-sided tumors. *POLE* mutations were mutually exclusive of dMMR and were associated with an upregulation in immune checkpoint genes and immunosuppressive proteins. In a study of 50 patients with resected stage II/III CRC with *POLE* mutations, both dMMR and *POLE* mutant tumors had less likelihood of recurrence, although *POLE* mutant tumors fared better with an 8% risk of recurrence compared to 18.9% among dMMR tumors and 27.8% among pMMR tumors, independent of *RAS* or *BRAF* status [75]. Another study by Stenzinger et al. analyzed 373 MSS CRC and discovered 54 *POLE* mutations in 46 samples, a higher incidence than previously reported (possibly due to more sensitive genotyping). OS and DFS were similar when comparing the *POLE* mutated cohort to nonmutated patients; however, stage III/IV disease treated with adjuvant or palliative chemotherapy had significantly worse survival compared to the wild-type cohort [78]. The predictive and prognostic implications of these findings have yet to be corroborated by a larger study. As a result, there are no guidelines available and routine testing in clinical practice is not recommended until prospective validation studies are complete.

## 4. Germline Pharmacogenomics

Table 3 summarizes key germline pharmacogenes and their clincial implication in CRC.

### 4.1. DPYD

Dihydropyrimidine dehydrogenase (DPD) is responsible for the catalytic metabolism of 5-FU into inactive metabolites. *DPYD*, the gene coding for DPD, is polymorphic and loss of enzyme activity can prolong 5-FU half-life by up to 100-fold [83]. Patients with DPD deficiency are at significantly higher toxicity risk, including severe neutropenia, diarrhea, and mucositis. Approximately 0.1% of all patients have no enzyme activity (i.e., are homozygous for a loss of function variant), while about 5–18% have an intermediate/deficient activity or are heterozygous for a reduced function allele, which is largely dependent on which SNPs are genotyped [84]. The most frequently studied variant is DPYD*2A with an allele frequency of only 1% in Caucasians [84].

Rosmarin et al. performed a systematic review in 927 colorectal cancer patients and demonstrated that global toxicity was significantly associated with DPYD*2A and the *c.2846T>A* SNP (combined OR 5.51; *p* = 0.0013) [85]. In a study of 2594 5-FU-treated colorectal cancer patients, Lee et al. observed the incidence of grade ≥ 3 toxicities in DPYD*2A, *I560S*, and *D949V* carriers were 22/25 (88.0%), 2/4 (50.0%), and 22/27 (81.5%), respectively. In a multivariate model, DPYD*2A (OR 15.21; *p* < 0.001) and *D949V* (OR 9.10; *p* < 0.001) were significantly associated with grade ≥ 3 toxicities [86]. Meulendijks et al. examined individual patient data from 7365 patients across 8 studies to investigate the association of DPYD*2A*, c.2846A>T*, *c.1679T>G*, *c.1236G>A/HapB3*, and c.1601G>A, with 5-FU-related toxicities. *DPYD c.1679T>G* (relative risk (RR) 4.40; *p* < 0.0001) and c.1236G>A/HapB3 (RR 1.59; *p* < 0.0001) were significantly associated with all grade 5-FU-related toxicity, whereas DPYD*2A and *c.2846A>T* were associated with severe overall toxicity (adjusted RR 2.85; *p* < 0.0001; and 3.02; *p* < 0.0001, respectively) [87]. 

In the largest prospective *DPYD* genotype-guided dosing study to date, Deenen et al. screened 2038 patients for DPYD*2A, of whom 22 were heterozygous and treated with an approximate 50% 5-FU dose reduction [88]. Grade ≥ 3 toxicity was reduced from 73% in historical controls (DPYD*2A carriers receiving full dose 5-FU) to 28% by genotype-guided dosing, while drug-induced death was reduced from 10% to 0%. 5-FU systemic drug exposure was similar between the two groups and the average total treatment cost per patient was lower for screening ($3767) than for non-screening ($3828), suggesting preemptive genotype-guided dose reductions improves tolerability with no increase in treatment cost [88]. More recently, Henricks et al. conducted a prospective study including all four major *DPYD* genotypes [89]. *DPYD* variant allele carriers received an initial dose reduction of 25% (*c.2846A>T* and *c.1236G>A)* or 50% (DPYD*2A and *c.1679T>G*). Of 1103 evaluable patients, 85 (8%) were heterozygous. Although the frequency of toxicities in variant carriers receiving dose reduction (39%) was significantly lower than historical control variant carriers receiving a full dose of fluoropyrimidine (>70%), the rate was still higher than wild-type patients receiving full dose (23%) (*p* = 0.0013) [89]. 

The Clinical Pharmacogenetics Implementation Consortium (CPIC) guidelines provide recommendations for dosing based on DPD enzyme activity or *DPYD* genotype: Patients with normal enzyme activity (*DPYD* wild-type) require no dose adjustment; patients with intermediate enzyme activity (heterozygous variant) require a 50% dose reduction, followed by titration of dose based on toxicity or exposure; and patients with complete enzyme deficiency (homozygous variant) should receive an alternative drug [79]. Based on the overwhelming evidence to date, preemptive screening for *DPYD* variants and respective dose adjustments should be incorporated into the FDA drug label and be established as the standard of care [90,91].

### 4.2. UGT1A1

Irinotecan, a topoisomerase I inhibitor, requires bioactivation to SN-38, which is 100–1000 times more potent than the parent drug. SN-38 is conjugated by uridine diphosphate glucuronosyltransferase 1A1 (UGT1A1) to SN-38G, which is inactive and excreted from the body [92]. The most common polymorphism in *UGT1A1* results in thymine-adenine (TA) repeats in the promoter region of the gene. TA repeats vary from 5 to 8, with 6 representing the normal phenotype. The wild type allele is referred to as UGT1A1*1, while the most common and studied variant, UGT1A1*28, results in 7 TA repeats and up to 70% reduction in enzyme activity (other less frequent variants include *6, *36, *37, *60, and *93). The frequency of the *28 allele is 39% in patients of European, 16% of Asian, and 43% of African origins [92].

A meta-analysis demonstrated a significant increase in grade 3/4 neutropenia for UGT1A1 *28/*28 patients at medium (150–250 mg/m^2^) (OR 3.22, *p* = 0.008) and high doses (>250 mg/m^2^) (OR 27.8, *p* = 0.005) [93]. Three genotype-guided phase I dose escalation trials in mCRC patients receiving FOLFIRI demonstrated that *1/*1 and *1/*28 patients can tolerate significantly higher doses than the standard FDA approved dose of 180 mg/m^2^. Toffoli et al. [94] found the maximum tolerated doses (MTDs) of irinotecan were 370 and 310 mg/m^2^ in *1/*1 and *1/*28, respectively and Marcuello et al. [95] identified MTDs of 390, 340, and 150 mg/m^2^ in *1/*1, *1/*28, and *28/*28, respectively. Both studies identified higher response rates and prolonged time to disease progression in patients treated at higher doses. Toffoli et al. performed another study in patients receiving FOLFIRI plus bevacizumab and identified MTDs of 310 and 260 mg/m^2^ in *1/*1 and *1/*28 [96]. A cost evaluation of irinotecan-related toxicities based on *UGT1A1* genotype in 243 mCRC patients identified the mean predicted cost per patient was higher for *28/*28 patients (€4886) compared to *1/*1 (€812) and *1/*28 patients (€1119) [97]. These findings are consistent with increased toxicity in *28/*28 patients and associated hospitalizations.

Presence of the *28/*28 genotype clearly increases toxicity risk and warrants dose reduction or close monitoring; however, it is relatively unknown whether administration of higher doses in *1/*1 and *1/*28 patients may result in improved survival outcomes. A phase II trial to answer this question is currently under investigation (Clinicaltrials.gov: NCT02138617). Studies have also shown that patients bearing polymorphisms in both *DPYD* and *UGT1A1* are at increased risk of experiencing clinically relevant toxicities, primarily hematological adverse events [98]. Given FOLFIRI is a first-line option in mCRC, it is prudent to consider preemptive genotyping to guide dosing of both 5-FU and irinotecan to reduce toxicity and optimize clinical outcomes. However, preemptive *UGT1A1* genotyping is not yet endorsed by most national guidelines even though it is mentioned in the FDA package insert as a risk factor for neutropenia. The Dutch Pharmacogenetics Working Group provides dosing guidelines based on the *28/*28 genotype, although this is only for situations where patients are receiving >250 mg/m^2^, which is above the standard dose recommended as part of the FOLFIRI regimen in first-line mCRC [80].

### 4.3. TYMS

Thymidylate synthase (TS) serves as the primary target of 5-FU. *TYMS*, the gene coding for TS, contains 28-base pair tandem repeat units found in the 5’ untranslated region (UTR), acting as an enhancer to the *TYMS* promoter [99]. Low *TYMS* expression may increase 5-FU cytotoxicity given reduced expression of the target and thereby lower threshold to inhibit pyrimidine biosynthesis, whereas high expression may decrease 5-FU cytotoxicity. Approximately 25% of European patients are homozygous for *TYMS 3R/3R*, which increases *TYMS* expression and 20% are homozygous for *2R/2R*, which decreases *TYMS* expression. A study of 927 capecitabine (oral 5-FU prodrug)-treated colorectal cancer patients from the QUASAR2 study identified global toxicity was associated with DPYD alleles *2846T>A* and *2A (OR 5.51; *p* = 0.0013) and with the *TYMS* polymorphisms *2R/3R* and 3’UTR 6bp insertion-deletion (OR 1.31; *p* = 9.4 × 10^‒6^) [85]. However, it is unknown whether preemptive *TYMS* genotyping is useful to guide fluoropyrimidine management until genotype-guided prospective randomized trial data are available. *TYMS* genotyping is not recommended in clinical practice and not endorsed by any guidelines.

### 4.4. MTHFR

Methylenetetrahydrofolate reductase (MTHFR) catalyzes the conversion of 5,10-methylenetetrahydrofolate (MTHF) to 5-methyltetrahydrofolate, the primary methyl donor for the re-methylation of homocysteine to methionine. A ternary complex is formed with FdUMP (active metabolite of 5-FU), TS, and 5,10-MTHF, which halts pyrimidine and DNA synthesis [100]. Thus, a reduction in MTHFR activity would increase 5,10-MTHF and increase 5-FU cytotoxicity.

In a study of 131 rectal cancer patients, *MTHFR 1298A>C* and *MTHFR* diplotypes (for *677C>T* and *1298A>C*) were associated with 5-FU chemoradiation-related toxicity, with no impact on drug response [100]; however, it is unknown the extent to which radiation played a role in the risk of toxicity. Another study of 117 advanced colorectal cancer patients receiving FOLFOX demonstrated no impact of *MTHFR* polymorphisms on toxicity; however, the additive score of *MTHFR* alleles *677T* and *1298C* was positively linked to response, with response rates of 37%, 53%, 63%, and 80.0% in patients bearing no, one, two, or three favorable alleles, respectively (*p* = 0.04) [101]. Due to the inconsistent findings, likely contributed to population and treatment heterogeneity, and lack of prospective studies, genotyping for MTHFR is not recommended in clinical practice and not endorsed by any guidelines. Large prospective validation studies, followed by genotype-guided trials, are required prior to consideration of implementation. 

### 4.5. ERCC

Nucleotide excision repair (NER) enzymes repair platinum-DNA adducts formed by administration of platinum analogs (e.g., oxaliplatin in FOLFOX). Mutations impairing the NER pathway can affect platinum sensitivity [102]. A meta-analysis of 1787 gastric and colorectal cancer patients treated with oxaliplatin-based regimens found that *ERCC1 rs11615C>T* was associated with reduced response and poor survival in Asians, whereas *ERCC2 rs13181T>G* was associated with reduced response and poor survival in Caucasians [102]. The largest study with a pre-planned analysis of *ERCC1* gene variants in colorectal cancer patients was the TOSCA trial, in which none of the studied polymorphisms showed association with toxicity or outcome [103]. Due to inconsistent findings and lack of prospectively validated data, genotyping for *ERCC1* or other NER genes is not recommended in clinical practice nor included on any guidelines. Large prospective validation studies, followed by genotype-guided trials, are required prior to consideration of implementation.

### 4.6. VEGF

VEGFA is an important regulator of angiogenesis in the VEGF family and has shown polymorphic potential [104]. In a study of 125 mCRC patients treated with FOLFIRI plus bevacizumab, rs833061 in *VEGFA* was significantly associated with response rate and PFS. Multivariate analyses demonstrated TT and TC genotypes of rs833061 were significantly correlated with enhanced therapeutic effects and prolonged PFS [104]. A meta-analysis of 1184 colorectal cancer patients also identified rs699947 and rs833061 SNPs (presence of at least one variant) were significantly associated with bevacizumab response; however, there was significant heterogeneity between studies, including treatments and population [105]. Other studies have suggested that VEGF polymorphisms may be associated with colorectal cancer prognosis due to its involvement with lymphangiogenesis and lymphatic tumor spread [106]. However, due to inconsistent findings and limited prospective studies, genotyping for VEGF polymorphisms is not recommended in clinical practice nor included on any guidelines. Large prospective validation studies, followed by genotype-guided trials, are required prior to consideration of implementation.

## 5. Conclusions

Identification of predictive and prognostic biomarkers are important for advancing and personalizing cancer treatments [107]. Although OS for mCRC has not drastically changed over the years, the discovery of physiological and molecular biomarkers has improved our ability to optimize treatment selection. Furthermore, germline pharmacogenomics may allow for better dose optimization and intensity. With the advancement in next generation sequencing technology and increased demand for molecular testing from both providers and patients, it is critical clinicians are aware of how to translate the presence of biomarkers into actionable prescribing decisions. Many institutions have implemented molecular tumor boards or personalized medicine consulting services to assist with detailed interpretation of molecular results for therapy/dose selection and clinical trial eligibility. The greatest impact may be in the refractory setting after the failure of first-line chemotherapy, where large scale sequencing is more commonly utilized to identify novel biomarkers. Additionally, the use of liquid biopsies (i.e., cell free DNA or circulating tumor DNA) may prove useful in disease monitoring, treatment response, and treatment selection in the refractory setting [108]. While dozens of potential biomarkers exist, it is critical to understand which are clinically validated and recommended for use in routine practice, as outlined in the tables. The ongoing discovery of predictive biomarkers will improve drug development of targeted therapies and immunotherapies that may substantially improve survival for CRC patients. 

## Figures and Tables

**Table 1:** Side-specific features in colorectal cancer (CRC).

Location	Right-Side (Appendix to Hepatic Flexure)	Left-Side (Splenic Flexure to Rectum)
Embryonic Origin	Midgut	Hindgut
Demographics	Female Older	Male ^a^ Younger ^a^
Prognosis	Worse prognosis in stage III + IV Worse response to EGFR inhibitors	Better survival in stage III + IV Better response to EGFR inhibitors
Cellular type	Goblet-like/mucinous Inflammatory CMS1 (MSI immune) CMS3 (metabolic) CCS2	Enterocyte Transit-amplifying CMS2 (canonical) CMS4 (mesenchymal) CCS1
Molecular Alterations	*BRAF* *dMMR* *EGFR*	*TP53* *APC* ^a^ *HER-2* ^a^
Microbiology	Predominant biofilms Increased fusobacterium	Rare biofilms

EGFR—epidermal growth factor receptor; CMS—consensus molecular subtypes; CCS—colon cancer subtype; BRAF—v-RAF murine sarcoma viral oncogene homolog B; APC—adenomatous polyposis coli; dMMR—deficient mismatch repair; HER2—human epidermal growth factor receptor 2; TP53—tumor protein 53. ^a^ More commonly noted in rectal cancer compared to colon cancer.

**Table 2:** Summary of somatic biomarkers and clinical implications in colorectal cancer.

Biomarker	Predictive, Prognostic, or both?	Frequency of Mutation in mCRC	Clinical Implication	Guideline Recommendation available?
*RAS*	Both	40% *KRAS* 3–5% *NRAS* <1% *HRAS*	Presence of RAS wild-type tumors allows for use of anti-EGFR monoclonal antibodies. Possible worse prognosis for mCRC liver-resection.	NCCN recommends extended *RAS* (K-, N- and H-) genomic testing for prognosis and treatment options [28].
*BRAF*	Both	8–12%	Portends worse OS. Possibly less benefit when treated with anti-EGFR monoclonal antibodies. Combination BRAF inhibition has poor response rates.	Recommend genomic testing; no treatment recommendations or guidelines available [28].
*MEK*	Both	Unknown	MEK targeting may require triple inhibition of *RAS*, *BRAF*, and *MEK*.	No treatment recommendations or guidelines available
*HER2*	Predictive	3–4% mCRC 5%*RAS* WT mCRC	Progression of *RAS* wild-type tumor with HER2 amplification may benefit from dual HER2 inhibition.	No treatment recommendations or guidelines available
MSI/dMMR	Both	15% of stage II–III 4–5% of stage IV	Prognosis is stage-dependent: Better in stage II and potentially worse in more advanced stages. Presence of MSI predictive of nonresponse to 5-FU but not validated. dMMR may predict an increased response to an immune checkpoint.	NCCN recommends MSI testing for patients with mCRC. It is also recommended in patients with high-risk for Lynch Syndrome and/or for prognosis determination [28].
PIK3CA	Potentially predictive	10–20% of all CRC	Better outcomes with post-operative aspirin use. Studies show possible resistance to anti-EGFR therapy, but this could be exon-specific	No treatment recommendations or guidelines available
TILs	PrognosticPredictive value is debated	No consensus due to the variability of measurement methods	Most studies show improved prognosis in all stages of CRC however large more uniform studies are necessary for consensus	No treatment recommendations or guidelines available
*POLE*	Prognostic and predictive values being investigated	0.65–12.3%	Variability in outcomes based on current studies. More responsive to immunotherapy in other tumor types but currently under investigation in CRC.	No treatment recommendations or guidelines available

RAS—rat sarcoma virus; KRAS—Kirsten rat sarcoma virus; EGFR—epidermal growth factor receptor; BRAF—v-RAF murine sarcoma viral oncogene homolog B; mCRC—metastatic colorectal cancer; OS—overall survival; MEK—mitogen activated protein kinase; HER2—human epidermal growth factor receptor; WT—wild type; MSI—microsatellite instability; dMMR—deficient mismatch repair; PIK3CA—phosphatidylinositol-4,5-bisphosphate 3-kinase catalytic subunit alpha; TIL—tumor infiltrating lymphocyte; POLE—polymerase epsilon, catalytic subunit.

**Table 3:** Summary of germline pharmacogenomic biomarkers and clinical implications in colorectal cancer.

Biomarker	Predictive, Prognostic, or both?	Frequency of Mutation ^a^	Clinical Implication	Guideline Recommendation Available?
*DPYD*	Predictive	Heterozygous: 5–8% Homozygous variant: <0.1%	Polymorphisms resulting in reduced DPD enzyme function significantly increase the risk of fluoropyrimidine-related toxicities and possibly death	- Dosing recommendations available from CPIC [79] and DPWG [80] - Mentioned under “Warnings and Precautions” in FDA package insert [81]
*UGT1A1*	Predictive	Heterozygous (*1/*28): 40–45% Homozygous variant (*28/*28): 10–15%	Polymorphisms resulting in reduced UGT1A1 activity significantly increase the risk of irinotecan-related toxicities, particularly at higher doses. Wild type patients may tolerate higher than standard doses.	- Dosing recommendations available from DPWG [80] - Mentioned under “Warnings and Precautions” in FDA package insert [82]
*TYMS*	Predictive	3R/3R: 25% 2R/2R: 20%	Low *TYMS* expression (2R/2R) may increase 5-FU cytotoxicity, whereas high expression (3R/3R) may decrease 5-FU cytotoxicity.	No treatment recommendations or guidelines available
*MTHFR*	Predictive	C677T MAF: 30–40% A1298C MAF: 30–35%	Reduction in MTHFR activity increases 5,10-MTHF and increases 5-FU cytotoxicity.	No treatment recommendations or guidelines available
*ERCC1/2*	Both	rs11615C>T MAF: 45–50% rs13181T>G MAF: 30%	Mutations impairing the NER pathway can render cancers more sensitive to platinum treatment.	No treatment recommendations or guidelines available
*VEGF*	Both	rs833061 MAF: 35–40% rs699947 MAF: 32–40%	Presence of *VEGFA* SNPs, which reduce expression, may improve bevacizumab response and survival outcomes.	No treatment recommendations or guidelines available

^a^ Allele or genotype frequencies are estimated for Caucasian or White populations. DPYD or DPD—dihydropyrimidine dehydrogenase; CPIC—Clinical Pharmacogenetics Implementation Consortium; DPWG—Dutch Pharmacogenetics Working Group; FDA—Food and Drug Administration; UGT1A1—uridine diphosphate glucuronosyltransferase 1A1; TYMS—thymidylate synthase; MTHFR—methylenetetrahydrofolate reductase; ERCC—excision repair complementing complex; NER—nucleotide excision repair; VEGF—vascular endothelial growth factor; MAF—minor allele frequency.
